# Robotic Cholecystectomy and Common Bile Duct Exploration: Multidisciplinary Approach in a Complex Case of Choledocholithiasis

**DOI:** 10.3390/jcm15176776

**Published:** 2026-08-31

**Authors:** Cataldo De Palma, Antonella Delvecchio, Miriam Varvara, Francesca D’Errico, Francesco Decembrino, Giuseppe Di Giovanni, Riccardo Inchingolo, Riccardo Memeo, Michele Tedeschi

**Affiliations:** 1 Unit of Hepato-Biliary and Pancreatic Surgery, Ecclesiastical Entity Regional General Hospital “F. Miulli”, Acquaviva delle Fonti, 70021 Bari, Italy; a.delvecchio@miulli.it (A.D.); r.memeo@miulli.it (R.M.); m.tedeschi@miulli.it (M.T.); 2 Department of Medicine and Surgery, LUM University, Parco il Baricentro 20B, Casamassima, 70010 Bari, Italy; r.inchingolo@miulli.it; 3 Unit of Anesthesia and Perioperative Medicine, Ecclesiastical Entity Regional General Hospital “F. Miulli”, Acquaviva delle Fonti, 70021 Bari, Italy; m.varvara@miulli.it; 4 Unit of Gastroenterology and Digestive Endoscopy, Ecclesiastical Entity Regional General Hospital “F. Miulli”, Acquaviva delle Fonti, 70021 Bari, Italy; f.derrico@miulli.it (F.D.); f.decembrino@miulli.it (F.D.); 5 Interventional Radiology Unit, Ecclesiastical Entity Regional General Hospital “F. Miulli”, Acquaviva delle Fonti, 70021 Bari, Italy; g.digiovanni@miulli.it

**Keywords:** robotic surgery, choledocholithiasis, common bile duct exploration

## Abstract

**Introduction:** Common bile duct (CBD) exploration is indicated in symptomatic patients with choledocholithiasis when endoscopic retrograde cholangiopancreatography (ERCP) is not feasible or has failed. In selected complex cases, robotic surgery may offer technical advantages over conventional laparoscopy. **Material and Methods:** A 51-year-old woman presented with biliary colic and ultrasound evidence of choledocholithiasis. Past medical history included congenital pyloric stenosis treated with open gastrojejunostomy at the age of 3 days, precluding ERCP. Magnetic resonance cholangiopancreatography (MRCP) and percutaneous transhepatic cholangiography (PTC) demonstrated multiple large distal CBD stones. Interventional radiology attempts failed to achieve complete clearance, and a percutaneous biliary drain was placed. The patient underwent robotic-assisted cholecystectomy and CBD exploration. **Results:** Following extensive adhesiolysis, robotic cholecystectomy was completed. A longitudinal choledochotomy enabled extraction of multiple stones using the chopstick technique, a Fogarty catheter, and choledochoscopic basket retrieval. After unsuccessful conventional retrieval attempts, an impacted stone was removed using a *choledochoscopy-guided robotic intraductal grasping maneuver*, with an 8-mm wristed forceps introduced through the choledochotomy and used exclusively for mechanical grasping. The choledochotomy was closed with continuous suturing, and intraoperative cholangiography confirmed unobstructed biliary flow without residual stones. The postoperative course was uneventful, and the patient was discharged on postoperative day seven. At two-month follow-up, cholangiography through the transcystic catheter confirmed persistent ductal clearance, allowing for catheter removal. **Conclusions:** This case demonstrates the feasibility of robotic CBD exploration in a selected patient with complex choledocholithiasis and altered anatomy when conventional treatment options were not feasible or unsuccessful.

## 1. Introduction

Choledocholithiasis affects approximately 5–20% of patients with symptomatic gallbladder stones and remains a common clinical challenge [1]. The prevalence has increased with the widespread use of advanced diagnostic modalities, particularly magnetic resonance cholangiopancreatography (MRCP) and endoscopic ultrasonography (EUS) [2,3]. The presence of common bile duct stones (CBDSs) can lead to severe complications, including obstructive jaundice, ascending cholangitis, hepatic abscess, and gallstone-related pancreatitis. Current guidelines indicate endoscopic retrograde cholangiopancreatography (ERCP) as the standard first-line therapeutic approach [4,5,6]. Innovative technical refinements in ERCP have expanded the available therapeutic options for stone clearance. These include endoscopic large balloon dilatation, mechanical lithotripsy, cholangioscopy-assisted lithotripsy, electrohydraulic lithotripsy, extracorporeal shock wave lithotripsy, and laser lithotripsy [3,4,5,6,7].

Surgically altered upper gastrointestinal anatomy represents an increasingly relevant challenge for biliary access, particularly in patients with previous gastrectomy, bariatric procedures, or gastrointestinal reconstruction, in whom conventional ERCP may be technically difficult or unfeasible [5,6]. In such settings, complex or impacted CBDSs may require alternative endoscopic, percutaneous, or surgical strategies, emphasizing the need for individualized multidisciplinary management.

Common bile duct exploration (CBDE) can be performed through an open approach or, when feasible, by minimally invasive techniques such as laparoscopic surgery or robotic surgery. When available, the minimally invasive approach is considered the preferred first-line surgical option, given its association with reduced morbidity and faster recovery [8].

Robotic-assisted choledochotomy was first reported in 2004 by Roeyen et al. [9], who demonstrated its feasibility in a single patient. Since then, accumulating evidence from case reports, small series and retrospective studies, has suggested that robotic-assisted CBDE is both safe and effective [10,11,12,13].

In selected complex cases, including patients with altered anatomy, dense adhesions from prior surgery, or large or impacted stones, the robotic approach may provide technical advantages over conventional laparoscopy, including superior visualization, tremor filtration, and enhanced dexterity.

We present a case of a female patient with complex choledocholithiasis and surgically altered anatomy, which was successfully managed with robotic cholecystectomy and CBD exploration within a multidisciplinary perioperative setting. In addition to illustrating the role of robotic CBDE when conventional biliary access is limited, this report describes a choledochoscopy-guided robotic intraductal grasping maneuver used as a rescue technique for an impacted stone after unsuccessful conventional basket retrieval. The present work was prepared in accordance with the CARE (CAse REport) guidelines (Supplementary File). The key clinical events are summarized chronologically in Table 1. A comprehensive overview of the clinical details and the surgical procedure, illustrated step by step, can be found in the *Video Abstract*.

## 2. Material and Methods

### 2.1. Case Presentation

A 51-year-old female presented to the emergency department with acute right upper quadrant abdominal pain associated with nausea and vomiting. Her past surgical history was significant for an open gastrojejunostomy performed at the age of 3 days for a congenital pyloric stenosis, a prior open repair of a median incisional hernia with mesh placement, and appendectomy. Her medical history included permanent atrial fibrillation, managed with direct oral anticoagulant (rivaroxaban 20 mg once daily) and β-blocker therapy (bisoprolol 2.5 mg once daily), and nodular thyroid disease with amiodarone-induced hypothyroidism. She also reported several previous episodes of cholangitis and a mild acute calculous pancreatitis, which were managed conservatively.

On presentation, laboratory evaluation showed no evidence of systemic inflammation, with a white blood cell count of 7230/mm^3^ [reference range: 4000–10,000/mm^3^] and C-reactive protein of 0.43 mg/dL [reference range: 0–0.50 mg/dL]. Liver tests showed elevated aspartate aminotransferase (AST, 156 U/L; reference range: 5–34 U/L), alanine aminotransferase (ALT, 166 U/L; reference range: 0–55 U/L), and gamma-glutamyl transpeptidase (GGT, 271 U/L; reference range: 0–38 U/L). Total bilirubin was mildly elevated (2.10 mg/dL; reference range: 0.20–1.20 mg/dL), with a direct bilirubin concentration of 0.64 mg/dL [reference range: 0–0.50 mg/dL]. Serum pancreatic amylase was 42 U/L [reference range: 8–51 U/L].

The clinical and laboratory findings were indicative of a symptomatic choledocholithiasis with biliary obstruction.

### 2.2. Imaging

The patient underwent abdominal ultrasonography, which demonstrated a distended gallbladder with regular wall thickness, dilation of both intrahepatic and extrahepatic bile ducts, and the presence of biliary sludge and stones within the CBD. Subsequent MRCP confirmed marked dilation of the entire biliary tree, with the CBD measuring up to 20 mm in diameter. Multiple lithiasic formations were observed within the distal CBD; the two largest stones measuring approximately 10 mm and 20 mm, respectively. Mild dilation of the main pancreatic duct was also noted, measuring 5 mm, likely secondary to choledocholithiasis. Additionally, the duodenum appeared ectatic with air-fluid levels and contained two prepapillary diverticula, measuring approximately 4 and 3 cm, respectively. The smaller diverticulum was located more anteriorly and in closer proximity to the major papilla.

### 2.3. Work-Up

Following the initial evaluation, esophagogastroduodenoscopy (EGD) was performed to assess endoscopic access to the biliary tree. EGD demonstrated a patent gastrojejunostomy, previously fashioned according to a Billroth II reconstruction, and severe, non-traversable pyloric stenosis. Although the afferent jejunal limb could be identified and traversed endoscopically, the major papilla could not be reached. Therefore, conventional ERCP was considered technically unfeasible.

Alternative endoscopic strategies were discussed. Although device-assisted enteroscopy-assisted ERCP (DAE-ERCP) was a potential option for the surgically altered anatomy, this technique was not available at our institution. EUS-guided biliary drainage with antegrade treatment was considered; however, this highly specialized approach was not pursued in this case given the complex and distorted anatomy and the available local expertise.

Percutaneous transhepatic cholangiography (PTC) was performed to further delineate the biliary anatomy and assess the stone burden. PTC confirmed multiple large stones within the distal common bile duct and partial obstruction of bile flow. Mechanical extraction using a Fogarty balloon was attempted but failed to achieve complete ductal clearance, and an internal-external biliary drain was placed to relieve cholestasis and maintain ductal patency.

Subsequent computed tomography (CT) confirmed the correct position of the percutaneous biliary drain, demonstrating partial regression of biliary dilatation but persistent distal CBDSs. No peritoneal fluid collections or other acute intra-abdominal findings were identified.

Considering the patient’s surgically altered and markedly distorted upper gastrointestinal anatomy, the technical impossibility of conventional ERCP, and the unsuccessful percutaneous mechanical extraction, the case was reviewed in a multidisciplinary setting involving hepatobiliary surgeons, interventional radiologists, endoscopists, and anesthesiologists. The multidisciplinary team considered either a two-stage strategy, including percutaneous cholangioscopy-guided electrohydraulic or laser lithotripsy through the existing transhepatic tract followed by delayed cholecystectomy, or a definitive single-stage surgical approach during the same admission. Consensus was reached for definitive surgical management via cholecystectomy and common bile duct exploration, with intraoperative planning aimed at achieving complete stone clearance while minimizing the risk of biliary injury.

### 2.4. Preoperative Surgical Plan

A preoperative surgical discussion was conducted to assess the optimal approach for the patient, taking into account her comorbidities. Given the patient’s permanent atrial fibrillation (CHA_2_DS_2_-VASc score: 1), chronic anticoagulation with rivaroxaban was discontinued 48 h before surgery. No preoperative heparin bridging was performed. Technical challenges related to prior abdominal surgeries were considered, including the presence of prosthetic mesh, the possibility of dense parietal and visceral adhesions, and the limited accessibility to the CBD due to the duodenal diverticula. In view of the aforementioned considerations, the decision was taken to employ a minimally invasive robotic-assisted procedure.

A stepwise operative strategy for the robotic CBDE was defined preoperatively. The planned key steps included: (1) patient positioning and insertion of robotic and assistant ports; (2) adhesiolysis and dissection of Calot’s triangle; (3) characterization of biliary stones using intraoperative ultrasound (IOUS); (4) choledochotomy in view of the stone size; (5) extraction of stones using appropriate techniques; (6) secure closure of the choledochotomy; and (7) intraoperative evaluation to confirm complete ductal clearance and absence of bile leak.

Perioperative antibiotic prophylaxis consisted of intravenous cefazolin (2 g) administered 60 min before skin incision and repeated after 4 h of surgery. Preoperative bile culture obtained through the percutaneous transhepatic biliary drain was negative.

## 3. Results

### 3.1. Patient Positioning and Insertion of Robotic and Assistant Ports

The procedure was conducted under general anesthesia. The patient was positioned supine in the reverse Trendelenburg position with split legs to adequately expose the supra-mesocolic compartment. The da Vinci XI robotic platform was employed, equipped with a 30° high-definition camera. Robotic trocars were inserted along the supraumbilical transverse line, while two additional assistant trocars were positioned in the infraumbilical region to facilitate retraction, suction, and instrument exchange.

### 3.2. Dissection of Calot’s Triangle

After induction of pneumoperitoneum, robotic docking was achieved. Dense adhesions from previous laparotomies were carefully lysed. The presence of duodenal diverticula firmly adherent to the hepatic hilum made access to the portal triad particularly challenging. Blunt dissection using Maryland dissector and bipolar forceps allowed for safe exposure of a markedly dilated cystic duct and CBD. The identification of Calot’s triangle was achieved through meticulous dissection of fibrotic inflammatory tissue, followed by antegrade mobilization of the gallbladder from the hepatic bed. Two cystic arteries, superior and inferior, originating from the right hepatic artery, were identified and divided between robotic Hem-o-lok clips.

### 3.3. Characterization of Biliary Stones Using IOUS

IOUS confirmed the presence of multiple large, impacted CBDSs, as well as the presence of the biliary drain. Doppler ultrasound imaging facilitated the identification of the anatomical relationships between the CBD, the portal vein, and the right hepatic artery, which coursed posterior to the bile duct.

### 3.4. Choledochotomy

Dissection proceeded distally along the cystic duct to its insertion into the CBD, which was located posteriorly. The anterior peritoneum covering the portal triad was opened and the CBD wall was identified. Gauzes and an endobag were adequately positioned to mitigate contamination and facilitate stone retrieval. The anterior wall of the CBD was opened vertically using robotic scissors, with care to avoid vascular injury at the 3 and 9 o’clock positions. An approximately 30-mm longitudinal choledochotomy was created in the midpoint of the CBD.

### 3.5. Extraction of Stones

**Figure 1:**
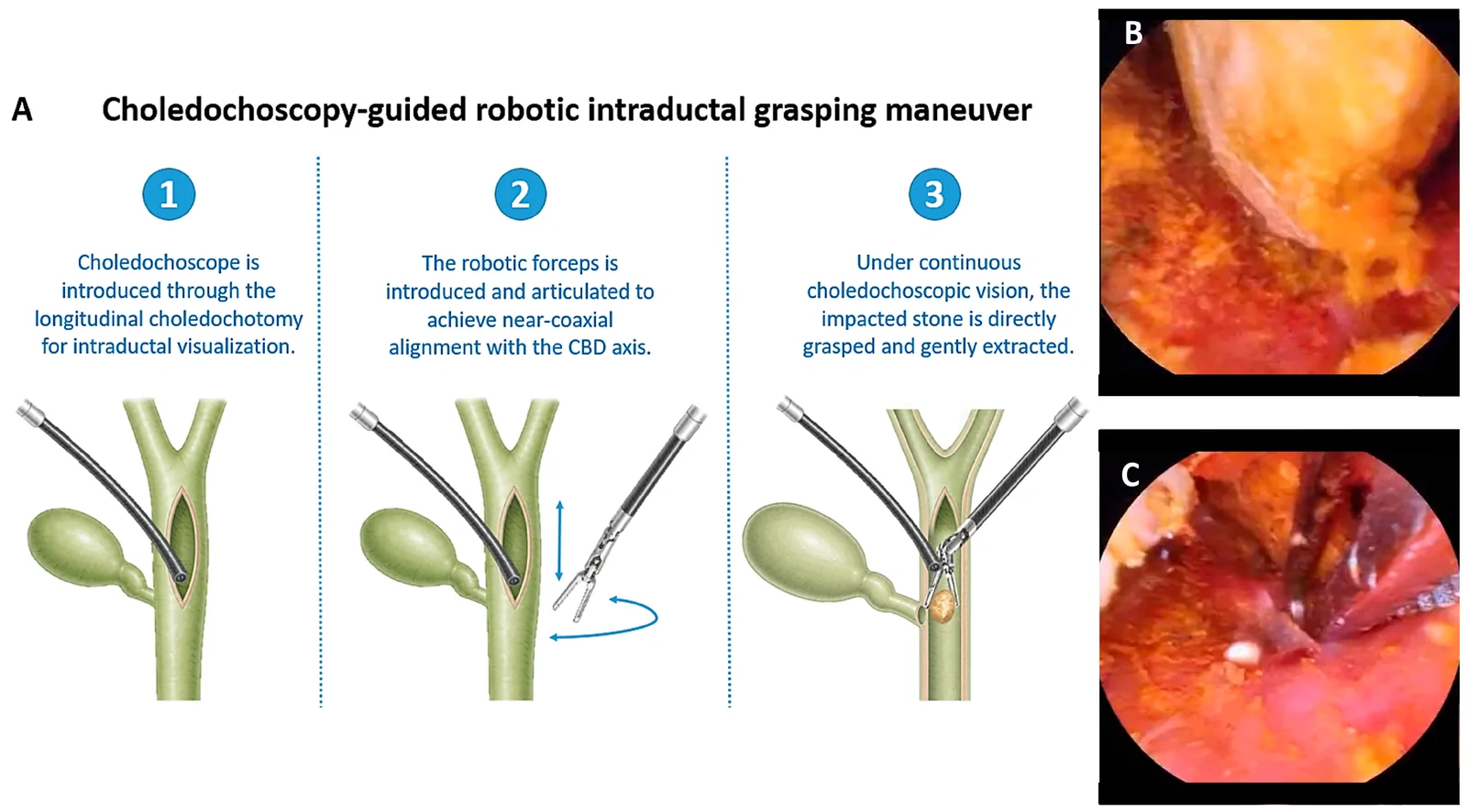
Choledochoscopy-guided robotic intraductal grasping maneuver. (**A**) Schematic representation of the three main steps of the maneuver. (**1**) The choledochoscope is introduced through the longitudinal choledochotomy to provide direct intraductal visualization. (**2**) The wristed robotic forceps is then introduced independently through the choledochotomy and articulated to achieve near-coaxial alignment of the jaws with the common bile duct (CBD) axis. The blue arrows indicate the direction of instrument articulation. (**3**) Under continuous choledochoscopic visualization, the impacted stone is directly grasped and gently extracted. (**B**) Choledochoscopic view showing the stone impacted at the cystic duct–CBD junction after unsuccessful conventional basket retrieval. (**C**) Direct grasping of the impacted stone with the 8-mm fenestrated bipolar forceps under continuous choledochoscopic visualization.

The *Chopstick technique* via choledochotomy was the primary approach. Gentle milking of the CBDSs from the distal to proximal direction was performed using the robotic forceps and the Maryland dissector. The first instrument was positioned on the right-posterior side to avoid injury to the portal vein, while the other was placed on the left-anterior side of the CBD. Two large stones were extracted through the choledochotomy site. The CBD was irrigated with 50 mL of saline solution via the catheter inserted from the assistant port to clear smaller stone fragments. IOUS revealed persistent residual stones. Another 5-mm assistant trocar was introduced in the right upper quadrant, and a collapsed 6 Fr Fogarty balloon catheter was inserted through the choledochotomy beyond the CBDSs under IOUS guidance. The balloon catheter was inflated with saline solution and continuously pulled back to extract the stones through the choledochotomy site. The percutaneous biliary drain was partially exteriorized to improve distal patency. Other biliary fragments were removed and the CBD lumen was once more irrigated. The subsequent IOUS revealed residual impacted stones. A 3.2-mm choledochoscope with a 1.2-mm working channel was introduced via the 5-mm auxiliary port through the choledochotomy using the free-drive technique under robotic guidance. Repeated retrieval attempts using a wire basket introduced through the working channel of the choledochoscope were unsuccessful because a 5-mm residual stone was firmly impacted at the cystic duct-CBD junction and could not be adequately captured by the basket. As a rescue maneuver, a pair of 8-mm da Vinci Xi Fenestrated Bipolar Forceps was used for direct intraductal mechanical retrieval. A schematic representation and representative intraoperative images illustrating the instrument configuration and direct choledochoscopic visualization during stone grasping are provided in Figure 1. The complete dynamic sequence of the maneuver is available in the accompanying *Video Abstract*. The wristed forceps was introduced independently through the approximately 30-mm longitudinal choledochotomy, articulated to achieve near-coaxial alignment with the CBD, and gently advanced into the ductal lumen under continuous direct choledochoscopic visualization. The jaws were kept closed during intraductal advancement and opened only after the instrument tip and the impacted stone were clearly visualized. The forceps was then used to obtain direct mechanical purchase on the exposed surface of the stone and to extract it through the choledochotomy. Bipolar energy was never activated while the instrument was within the bile duct, thereby avoiding energy-related thermal injury. The forceps was used exclusively as a mechanical grasper. Grasping was directed exclusively at the stone, under continuous choledochoscopic visualization, and slow, controlled traction was applied without blind sweeping, forceful rotation, or excessive lateral traction. The length of the choledochotomy provided adequate access for controlled introduction of the robotic instrument without requiring forced dilation of the ductal opening. The wristed articulation was used to maintain near-coaxial alignment with the CBD and minimize levering against the choledochotomy edges and lateral ductal wall. Following extraction, direct choledochoscopic inspection showed no evidence of ductal wall injury, perforation or bleeding and confirmed complete ductal clearance. Intraoperative electrohydraulic or laser lithotripsy was not necessary.

### 3.6. Closure of the Choledochotomy

The cystic duct was divided, and cholecystectomy was completed. The transhepatic biliary drain, which had been exteriorized through the choledochotomy during duct exploration, was removed because the distal pigtail anchoring mechanism could no longer be reliably re-established within the duodenum. A 6-Fr transcystic Bracci catheter was placed to provide temporary biliary decompression and postoperative cholangiographic access, as postoperative endoscopic access to the biliary tree was not feasible. The catheter was secured with an absorbable suture. The longitudinal choledochotomy was primarily closed with a continuous 3-0 absorbable barbed V-Loc suture, chosen to allow for controlled intracorporeal suturing and uniform approximation of the choledochotomy edges.

### 3.7. Intraoperative Evaluation to Confirm Complete Ductal Clearance

C-Arm fluoroscopy was prepared and intraoperative cholangiography was performed by injecting 10–15 mL of diluted water-soluble contrast through the Bracci catheter. Dynamic fluoroscopy confirmed free flow into the duodenum, with no evidence of residual stones, leakage, or obstruction.

Hemostasis was verified, a Jackson–Pratt drain was positioned in the subhepatic space, and the specimen was retrieved in an endobag. Robotic undocking was performed, and fascial and skin closure was completed. The total operative time was 430 min, with an estimated blood loss of 100 mL.

### 3.8. Post-Operative Course

The postoperative course was uneventful. Monitoring included serial clinical assessment, laboratory evaluation of inflammatory markers, hemoglobin and liver function tests, and daily assessment of the subhepatic drain output and characteristics. The patient remained afebrile throughout the postoperative course. The subhepatic Jackson–Pratt drain yielded approximately 100 mL of serosanguineous fluid on postoperative day (POD) 1, followed by a progressive decrease in output, which became serous without evidence of bilious drainage. Drain-fluid bilirubin was not measured because there was no clinical suspicion of bile leakage. C-reactive protein reached a postoperative peak of 7.2 mg/dL on POD3 and subsequently progressively decreased. The transcystic catheter was closed on POD5. Following abdominal ultrasonography showing no evidence of intra-abdominal fluid collections, the Jackson–Pratt drain was removed on POD7.

Prophylactic enoxaparin (4000 IU once daily) was started on POD1 and continued through POD3, after which rivaroxaban 20 mg once daily was resumed. No postoperative bleeding or thromboembolic events occurred. Preoperative hemoglobin was 12.1 g/dL (reference range, 12.0–16.0 g/dL) and was 12.7 g/dL at discharge; no blood transfusion was required. Oral intake was promptly resumed, liver function tests progressively normalized, and the patient was discharged on POD7 in good clinical condition.

At the two-month follow-up, cholangiography performed through the transcystic catheter confirmed persistent complete ductal clearance and unobstructed flow of contrast into the duodenum, with no evidence of biliary obstruction or leakage. The catheter was subsequently removed without complications.

## 4. Discussion

The present case illustrates the role of robotic surgery in managing complex and difficult choledocholithiasis in a patient with surgically altered upper gastrointestinal anatomy. Conventional ERCP was technically unfeasible because the major papilla could not be reached, despite endoscopic identification and traversal of the afferent limb, in the setting of a Billroth II-type reconstruction, non-traversable pyloric stenosis, and markedly distorted duodenal anatomy. DAE-ERCP represented a potential alternative for the surgically altered anatomy but was not available, while EUS-guided biliary drainage with antegrade treatment was not pursued because of the complex anatomy and available local expertise. Following unsuccessful percutaneous mechanical stone extraction, the multidisciplinary discussion focused on percutaneous cholangioscopy-guided lithotripsy, followed by delayed cholecystectomy, versus definitive single-stage surgical treatment. The latter was selected to address both the gallbladder and CBD stones during the same procedure. Given the institutional expertise in robotic surgery, this approach was chosen to facilitate precise dissection and biliary exploration in this complex anatomical setting through enhanced dexterity, three-dimensional visualization, and tremor filtration. The total operative time was 430 min and should be interpreted in the context of the exceptional technical complexity of this case rather than as an expected duration of robotic CBDE. The procedure required extensive adhesiolysis following previous open abdominal operations, careful dissection of large duodenal diverticula that were densely adherent to the hepatic hilum, and management of markedly distorted biliary anatomy. In addition, several sequential intraoperative modalities were required to achieve and confirm ductal clearance, including IOUS, external ductal manipulation, irrigation, Fogarty balloon extraction, choledochoscopy with repeated basket retrieval attempts, robotic intraductal grasping, primary closure of the choledochotomy, and intraoperative cholangiography. Complete ductal clearance was defined intraoperatively by concordant findings from direct choledochoscopy, IOUS, and cholangiography. Specifically, final choledochoscopy demonstrated no visible residual stones or fragments, IOUS showed no residual intraductal stones, and cholangiography demonstrated no persistent intraluminal filling defects together with free passage of contrast into the duodenum. Cholangiography additionally confirmed the absence of contrast extravasation from the biliary tree. The complete sequence of preoperative imaging, intraoperative assessment, stone extraction, and confirmation of ductal clearance is documented in the accompanying *Video Abstract*. The patient experienced an uneventful recovery. These outcomes can be attributed to careful preoperative planning and effective multidisciplinary collaboration, both pre- and intraoperatively [3].

Currently, there is no standardized protocol for selecting robotic CBDE. However, guidelines recommend surgical CBDE in cases defined as “difficult CBDSs” because it remains a rescue treatment when endoscopic attempts fail [4,5,6]. ESGE guidelines define “difficult CBDSs” as stones with a diameter > 1.5 cm, multiple or barrel-shaped stones, an intrahepatic or cystic duct location, impaction, altered anatomy, distal bile duct angulation <135°, sigmoid-shaped CBD, and short-length ducts [5]. Two conventional surgical approaches exist for CBDE: the transcystic (TC) and transductal (TD) routes. Larger stones (>6 mm), small cystic ducts (<4 mm), intrahepatic stones, and distal or posterior cystic duct insertion into the CBD are associated with poor TC success rates, favoring the TD approach [14].

Minimally invasive CBDE has been explored predominantly through studies assessing the laparoscopic approach, which have evidenced its feasibility and safety [8]. However, laparoscopic techniques present limitations, including two-dimensional vision, lack of depth perception, reduced degrees of freedom of instruments, and the fulcrum effect, which creates a disparity between visual feedback and proprioceptive function [15]. Robotic-assisted choledochotomy and CBDE may overcome many of these disadvantages. In our experience, the robotic system allowed complex intraoperative maneuvers for CBD exploration, such as the *choledochoscopy-guided robotic intraductal grasping maneuver*, which is not otherwise feasible without articulating instruments. Previous descriptions of robotic common bile duct exploration have reported choledochoscopy-assisted stone extraction, predominantly using retrieval baskets introduced through the working channel of the choledochoscope, as well as lithotripsy followed by flushing or basket retrieval [10,12]. In the present case, repeated choledochoscopic basket attempts failed because the residual stone was firmly impacted at the cystic duct–CBD junction and could not be adequately captured. The rescue maneuver used in this case differs technically from conventional choledochoscopic basket retrieval. The 3.2-mm choledochoscope, with its 1.2-mm working channel, was used to provide continuous intraductal visualization, whereas an 8-mm wristed robotic Fenestrated Bipolar Forceps was introduced independently through the approximately 30-mm choledochotomy. The forceps was articulated to achieve near-coaxial alignment with the CBD and advanced under direct choledochoscopic control. This configuration allowed the instrument to obtain direct mechanical purchase on the exposed surface of the impacted stone without requiring circumferential deployment of a basket around it. Bipolar energy was never activated during intraductal manipulation. Given the successful clearance obtained mechanically, the intraoperative choledochoscopy-guided lithotripsy was not necessary.

To the best of our knowledge, we found no previous description of direct intraductal extraction of an impacted CBD stone using wristed robotic forceps introduced independently through a choledochotomy under simultaneous direct choledochoscopic visualization. We therefore describe this as a choledochoscopy-guided robotic intraductal grasping maneuver and consider it a potentially useful rescue option in selected impacted stones that cannot be captured by conventional basket retrieval. Nevertheless, its safety, reproducibility, and potential advantages over established retrieval or lithotripsy techniques cannot be established from a single case and require further evaluation. Despite the benefits of robotic surgery, the concern of elevated costs remains. Almamar et al. [16] compared robotic and open CBDE, finding no significant difference in operative time but demonstrating lower postoperative complications and shorter hospital stays (6 vs. 12 days). Notably, total hospital costs were lower in the robotic group despite higher procedural expenses, underscoring potential cost-effectiveness in selected complex cases.

CBDSs in patients with surgically altered anatomy, such as after gastrectomy with Billroth I/II reconstruction, esophagectomy with gastric conduit, pancreaticoduodenectomy, hepaticojejunostomy, or Roux-en-Y gastric bypass, represent a significant challenge for endoscopic therapy [6]. While specialized techniques have been developed by experienced endoscopists, they are not universally available, leaving surgery as the only viable option in certain cases. Prior upper abdominal surgery is significantly associated with dense adhesions and distortion of Calot’s triangle. Although minimally invasive CBDE is feasible in such patients, the increased technical difficulty often results in a higher conversion rate to open surgery [17]. In the study conducted by Huang et al. [18], patients with prior upper abdominal surgery undergoing laparoscopic CBDE experienced longer operative times, increased costs, and higher conversion rates. Similarly, Li et al. [19] reported a 17% conversion rate in patients with prior biliary surgery, highlighting the impact of adhesions and distorted anatomy on procedural complexity.

Although the current evidence remains limited and is predominantly based on retrospective series and observational studies, available data suggest that robotic CBDE is technically feasible in selected patients, with encouraging perioperative and stone-clearance outcomes. A recent meta-analysis [20] that included 438 patients who underwent robotic CBDE reported an overall pooled stone clearance rate of 95% and an overall pooled complication rate of 25%. Comparative analysis showed that robotic CBDE was associated with a 34% lower risk of complications compared to open surgery, although open procedures demonstrated a shorter operative time, a difference of 38 min on average. The multicentric study by Stefanova et al. [21], which included 102 patients, revealed that performing robotic CBDE in conjunction with cholecystectomy resulted in a significantly lower conversion rate compared to isolated CBDE in patients with prior biliary surgery (1.4% vs. 30.4%). Further prospective studies are needed to draw definitive conclusions regarding robotic CBDE safety and efficacy compared with other treatments or surgical approaches.

In order to reduce the risk of postoperative bile leakage after choledochotomy in transductal CBDE, intraductal decompression could be considered a valuable strategy, especially in cases where prior sphincterotomy has not been performed [8]. Routine T-tube drainage, once standard, is now largely discouraged due to its association with higher operative times and hospital stay, patient discomfort, dislodgement, infection, electrolyte imbalance, and bile peritonitis [22,23]. Instead, antegrade stent placement has advantages in reducing hospital stay and post-operative complications compared to T-tubes [24,25].

In the present case, a transcystic drain was preferred to reduce intraluminal pressure and minimize the risk of postoperative fistula, as antegrade stent placement was not feasible nor manageable postoperatively due to an endoscopically impassable pyloric stenosis. The drain was maintained closed to prevent electrolyte loss, irrigated daily to preserve patency, and provided both postoperative cholangiographic monitoring and a potential access route for interventional radiology in case of recurrence. No postoperative complications occurred.

The occurrence of stricture after CBDE is uncommon, with a reported incidence ranging between 0% and 0.8% [6]. In the TD approach, preservation of the CBD blood supply is paramount, as ischemic injury from interruption of arteriolar branches is a recognized cause of stricture [26]. Choledochotomy can be performed either longitudinally or transversely [27,28]. Paganini et al. [29,30] recommend transverse choledochotomy to reduce the risk of interrupting marginal arterioles, although longitudinal choledochotomy provides greater flexibility for extension in cases of large stones and facilitates choledochoscope insertion [31].

In the present case, a longitudinal choledochotomy was selected because of the markedly dilated CBD, the presence of large and impacted stones measuring up to approximately 20 mm, and the need for adequate access for repeated stone-retrieval maneuvers and choledochoscopy. The approximately 30-mm longitudinal incision also permitted controlled introduction of the 8-mm wristed robotic forceps during the rescue intraductal grasping maneuver without forced dilation of the ductal opening. This technical advantage was balanced against the potential vascular concern associated with a longitudinal incision by maintaining the choledochotomy on the anterior aspect of the CBD and avoiding extension or excessive dissection toward the lateral 3- and 9-o’clock margins, where the longitudinal arterial supply is particularly vulnerable. No randomized evidence currently establishes the superiority of either incision orientation, and the choice should therefore be individualized according to duct diameter, stone characteristics, required intraductal maneuvers, and local anatomy.

Although the two-month follow-up confirmed ductal clearance and an uncomplicated early postoperative outcome, this interval is insufficient to assess late biliary complications, particularly post-choledochotomy stricture and recurrent choledocholithiasis. Longer clinical and imaging follow-up is therefore required to evaluate the durability of the ductal patency and stone clearance.

Robotic CBDE should ideally be performed in high-volume centers by surgeons experienced in minimally invasive and hepatobiliary surgery, with comprehensive knowledge of biliary anatomy, proficiency in intraoperative cholangiography and IOUS, advanced intracorporeal suturing skills, and expertise in various stone extraction techniques, including the Chopstick method, balloon and Dormia basket retrieval, and choledochoscopy-assisted removal [5,6,7,8].

## 5. Conclusions

ERCP remains the first-line approach for the management of CBDSs. Nevertheless, surgical CBDE remains indicated in selected patients with difficult stones, unsuccessful endoscopic treatment, or altered anatomy precluding conventional endoscopic access. This case demonstrates the technical feasibility of robotic CBDE in a selected patient with complex choledocholithiasis and surgically altered anatomy. The *choledochoscopy-guided robotic intraductal grasping maneuver* provided a rescue option for an impacted stone after unsuccessful conventional retrieval in this specific case. However, the findings of a single case cannot establish the safety, efficacy, reproducibility, or comparative benefits of either the maneuver or robotic CBDE more broadly. Such procedures should be considered in appropriately selected patients at experienced centers with expertise in hepatobiliary and minimally invasive surgery and within a multidisciplinary setting. Further studies are required to establish the safety, efficacy, and comparative benefits of the robotic approach.

## Figures and Tables

**Table 1:** Case Timeline.

Time Point	Event
**Neonatal period**	Congenital pyloric stenosis → Billroth II-fashioned gastrojejunostomy
**Presentation**	Symptomatic choledocholithiasis with biliary obstruction
**Diagnostic work-up**	US, MRCP and EGD → major papilla not reachable → ERCP not feasible
**Preoperative treatment**	PTC → failed Fogarty-balloon extraction → PTBD placement
**Surgery**	Robotic cholecystectomy, choledochotomy and CBDE **Stone extraction techniques**: chopstick maneuver, flushing, Fogarty balloon, choledochoscope-guided basket retrieval, robotic intraductal grasping maneuver **Clearance confirmation**: IOUS, choledochoscopy and cholangiography
**POD 5**	Transcystic catheter closure
**POD 7**	Abdominal US → JP drain removal → discharge
**2 months**	Transcystic cholangiography → catheter removal

**Abbreviations**: CBDE: common bile duct exploration; EGD: esophagogastroduodenoscopy; ERCP: endoscopic retrograde cholangiopancreatography; IOUS: intraoperative ultrasonography; JP: Jackson–Pratt; MRCP: magnetic resonance cholangiopancreatography; POD: postoperative day; PTC: percutaneous transhepatic cholangiography; PTBD: percutaneous transhepatic biliary drainage; US: ultrasonography.

## Data Availability

All data generated or analyzed during this study are included in this published article. Further methodological details are available from the corresponding author upon reasonable request.

## Supplementary Materials

The following supporting information can be downloaded at: https://www.mdpi.com/article/10.3390/jcm15176776/s1, Supplementary File: CARE Checklist.
