# Supplementary material for: Robotic Cholecystectomy and Common Bile Duct Exploration: Multidisciplinary Approach in a Complex Case of Choledocholithiasis
Source: J Clin Med. 2026 Aug 31;15(17):6776. doi: 10.3390/jcm15176776 (PMC13566010; doi:10.3390/jcm15176776)
Supplement: Supplementary file 1 [file jcm-15-06776-s001.zip › jcm-4440446-supplementary.pdf]

CARE case report guidelines

CARE Checklist of information to include when writing a case report

(cc) BY-NC-ND

Topic	Item	Checklist item description	Reported on Line
**Title**	**1**	The diagnosis or intervention of primary focus followed by the words "case report"	Page 1 Lines 2-4
**Key Words**	**2**	2 to 5 key words that identify diagnoses or interventions in this case report, including "case report"	Page 2 Line 43
**Abstract (no references)**	**3a**	Introduction: What is unique about this case and what does it add to the scientific literature?	Page 1 Lines 19-21, Lines 32-35
	**3b**	Main symptoms and/or important clinical findings	Page 1 Lines 22-25
	**3c**	The main diagnoses, therapeutic interventions, and outcomes	Page 1 Lines 25-39
	**3d**	Conclusion—What is the main "take-away" lesson(s) from this case?	Page 1 Lines 39-42
**Introduction**	**4**	One or two paragraphs summarizing why this case is unique (**may include references**)	Page 2 Lines 58-63, 77-86
**Patient Information**	**5a**	De-identified patient specific information.	Page 3 Lines 94-102
	**5b**	Primary concerns and symptoms of the patient.	Page 3 Lines 94-95
	**5c**	Medical, family, and psycho-social history including relevant genetic information	Page 3 Lines 95-102
	**5d**	Relevant past interventions with outcomes	Page 3 Lines 95-102
**Clinical Findings**	**6**	Describe significant physical examination (PE) and important clinical findings.	Page 3 Lines 94-102
**Timeline**	**7**	Historical and current information from this episode of care organized as a timeline	Page 2 Line 84, Table 1
**Diagnostic Assessment**	**8a**	Diagnostic testing (such as PE, laboratory testing, imaging, surveys).	Page 3 Lines 103-113 / Page 4 Lines 114-124
	**8b**	Diagnostic challenges (such as access to testing, financial, or cultural)	Not applicable
	**8c**	Diagnosis (including other diagnoses considered)	Page 3 Lines 103-113 / Page 4 Lines 114-124
	**8d**	Prognosis (such as staging in oncology) where applicable	Not applicable
**Therapeutic Intervention**	**9a**	Types of therapeutic intervention (such as pharmacologic, surgical, preventive, self-care)	Page 4 Lines 125-158
	**9b**	Administration of therapeutic intervention (such as dosage, strength, duration)	Page 4 Lines 159-161; Pages 5-7; Page 8 Lines 279-280
	**9c**	Changes in therapeutic intervention (with rationale)	Page 4 Lines 125-158
**Follow-up and Outcomes**	**10a**	Clinician and patient-assessed outcomes (if available)	Page 8 Lines 281-302
	**10b**	Important follow-up diagnostic and other test results	Page 8 Lines 281-302
	**10c**	Intervention adherence and tolerability (How was this assessed?)	Page 8 Lines 281-302

	**10d**	Adverse and unanticipated events	Page 8 Lines 281-302
**Discussion**	**11a**	A scientific discussion of the strengths AND limitations associated with this case report	Page 8 Lines 303-327 / Pages 9-10; Page 11 Lines 430-459
	**11b**	Discussion of the relevant medical literature **with references**.	Page 8 Lines 303-327 / Pages 9-10; Page 11 Lines 430-459
	**11c**	The scientific rationale for any conclusions (including assessment of possible causes)	Page 11 Lines 454-459
	**11d**	The primary "take-away" lessons of this case report (without references) in a one paragraph conclusion	Page 11 Lines 460-472
**Patient Perspective**	**12**	The patient should share their perspective in one to two paragraphs on the treatment(s) they received	Not applicable
**Informed Consent**	**13**	Did the patient give informed consent? Please provide if requested	**Yes** X **No** ☐
